# Surgical excision of paraurethral cyst

**DOI:** 10.1590/S1677-5538.IBJU.2018.0761

**Published:** 2020-01-10

**Authors:** Rodolfo Milani, Stefano Manodoro, Alice Cola, Stefania Palmieri, Claudio Reato, Matteo Frigerio

**Affiliations:** 1 Department Ginecologia Chirurgica San Gerardo Hospital Monza Italy Department Ginecologia Chirurgica, San Gerardo Hospital, Monza, Italy;; 2 AUSL Romagna Infermi Hospital Rimini Italy AUSL Romagna, Infermi Hospital, Rimini, Italy

## Abstract

**Purpose:**

Patients with paraurethral cyst may be asymptomatic or bothered by sensation of a mass, pain, distorted urinary outflow, dyspareunia, and dysuria (1). Differential diagnosis includes ectopic ureterocele, pelvic organ prolapse, and urethral diverticulum. At present, the management of paraurethral cysts is unclear, but surgical excision appears as the best treatment option (1-3). Alternative methods include waiting for spontaneous rupture, needle aspiration and marsupialization (4). The aim of the video-tutorial is to provide anatomic views and surgical steps necessary to achieve a successful complete excision of a paraurethral cyst.

**Materials and methods:**

A 54-year-old woman with a 2cm paraurethral cyst bothered by intermittent sensation of an introital mass, dyspareunia, and dysuria was admitted to surgical excision according to the described technique. Urethrocystoscopy and ultrasonography were preoperatively performed to confirm the diagnosis and rule out an urethral diverticulum. Surgical steps included: cyst exposure; vaginal mucosa incision; adequate dissection (needle injection of saline solution inside the cyst can be performed to inflate the cyst) with scissors and swab, isolation and excision of paraurethral cyst, layered reconstruction with avoidance of suture layers overlapping.

**Results:**

Surgical procedure was successfully achieved without complications. The postoperative course was uneventful. No recurrence was observed and the patient reported complete resolution of her symptoms.

**Conclusions:**

The featured video showed complete excision of a paraurethral cyst successfully achieved without complications. Surgical excision represents a safe and effective procedure to manage paraurethral cysts. This step-by-step video-tutorial may represent an important tool to improve surgical know-how.

Available at: http://www.intbrazjurol.com.br/video-section/20180761_Milani_et_al
